# Virtual Intergenerational Reverse-Mentoring Program Reduces Loneliness among Older Adults: Results from a Pilot Evaluation

**DOI:** 10.3390/ijerph19127121

**Published:** 2022-06-10

**Authors:** Jill J. Juris, Erin D. Bouldin, Katherine Uva, Christopher D. Cardwell, Anastacia Schulhoff, Nicole Hiegl

**Affiliations:** 1 Beaver College of Health Sciences, Appalachian State University, Boone, NC 28608, USA; erin.bouldin@hsc.utah.edu (E.D.B.); uvakr@appstate.edu (K.U.); ccardwell@msahealthcare.com (C.D.C.); 2 College of Arts and Sciences, Appalachian State University, Boone, NC 28608, USA; schulhoffam@appstate.edu; 3 High Country Area Agency on Aging, Boone, NC 28607, USA; nicolehiegl@gmail.com

**Keywords:** intergenerational, loneliness, social isolation, program evaluation, internet-based intervention, rural population, reverse mentoring

## Abstract

Social isolation and loneliness can create negative health outcomes for older adults. Informed by social capital and intergroup contact theories, our goal was to reduce these social problems using an intergenerational reverse-mentoring program. During fall 2020, we implemented an adapted, fully online version of Cyber-Seniors that encouraged undergraduate students to provide technology mentoring to local older adults in a seven-county area in rural Appalachia. We recruited gerontology students through the university and local older adults through local aging organizations. We collected data through pre-and post-tests that included validated measures (Lubben Social Network Scale-6 and UCLA 3-item loneliness scale) and open-ended questions about the program. Thirty-one students and nine older adults completed the pre-survey; twenty students and eight older adults completed the post-survey. We made comparisons using t-tests and considered *p* < 0.20 to indicate meaningful differences given the anticipated small sample size in this pilot project. Isolation did not change among older adults but increased among students in the family domain (*p* = 0.14) between baseline and follow-up. Loneliness improved between the pre- and post-tests among older adults (mean: 5.6 (SD = 2.2) to 4.1 (SD = 1.3), *p* = 0.17) but not among students (mean: 5.0 (SD = 1.5) to 5.2 (SD = 1.7), *p* = 0.73). In open-ended responses, older adults described learning new ways to interact with friends and family as a result of the program. This program was acceptable and suggested effectiveness in an important health-related domain (loneliness). While larger studies are needed to fully test the program’s impact, this pilot evaluation suggests that reverse mentoring programs can be implemented virtually and may improve social outcomes.

## 1. Introduction

Social isolation and loneliness can create negative health outcomes for older adults [1,2]. Increasing evidence shows that older adults’ social connections significantly reduce the risk of death. Holt-Lundstad and colleagues found that greater social connection is associated with a 50% reduced risk of mortality [1]. More specifically, lacking social connections confers a greater risk of mortality than risk factors such as air pollution, obesity, and smoking up to 15 cigarettes per day [1]. In the longitudinal Health and Retirement Study, respondents over 60 years old who experienced loneliness had a decline in activities of daily living and mobility, indicating functional decline and an increased risk of death [3]. As Americans are becoming less socially connected, they are at increased risk for depression, dementia, and cognitive decline [4]. One study found that among community-dwelling older adults living in West Virginia, which is part of the Appalachian region of the United States, social isolation was associated with poorer cognitive function overall and with the domains of memory, executive function, and attention specifically [5]. Social isolation refers to having a limited social support network of people one can rely on for emotional and practical support [6]. Loneliness is a subjective feeling of isolation due to a difference between desired and actual levels of connection with others [3,7]. Social connections can be linked to health outcomes using social capital theory, which posits that social connections lead to greater resources to successfully navigate the life course [8]. Social relationships promote better health among older adults by increasing a sense of belonging, creating emotional support networks, and providing opportunities for transferring information (e.g., treatments, medications) [1,9].

Prior to COVID-19, loneliness was identified as an epidemic, and public health efforts to increase social connections were prioritized [10]. COVID-19 certainly created challenges to social interaction because of stay-at-home orders and fears of becoming infected, which, in turn, led to increased social isolation and loneliness [11,12,13]. Teater and colleagues conducted a cross-sectional study to explore social isolation and loneliness levels among young, middle-aged, and older U.S. adults before and during COVID-19 [14]. They found U.S. older adults experienced significant increases in social isolation and loneliness. Additionally, they found that during COVID-19, there was a positive correlation between older adults reporting social isolation and feeling lonely, including emotional loneliness, social loneliness, and overall general loneliness [14].

In order to address isolation and loneliness during COVID-19, many people in the U.S. turned to digital technologies [2]. These technologies facilitated communication and connection when people could not be physically present with others or have face-to-face healthcare visits [15]. However, older adults less frequently use technology because of differences in access to computers and the internet [2,16] and differences in literacy to utilize online platforms to access the same systems [17] compared to younger adults. During the pandemic, 74% of Americans 65 years and older communicated with others through email and/or messaging platforms compared to 80% of people under age 50 [18]. This difference is more pronounced in rural areas that lack broadband access [2]. Within the Appalachian region of the United States, 71% of households in rural counties have internet subscriptions, compared to 83% of households across the United States [19].

One approach to reducing the generational divide is an intergenerational technology program [20]. Intergenerational programs consist of opportunities for unrelated younger (usually 24 years old and under) and older (usually 50 years old and over) persons to interact in meaningful activities [21]. Intergenerational programs bring disparate age groups together for mutual benefit. Allport established the intergroup contact theory describing how two disparate groups can come together for positive interactions [22], and this theory has been applied in online settings [23]. Intergenerational studies that bring together two disparate age groups in community settings are often guided by the tenets of contact theory [24,25,26]. Intergroup contact theory posits five tenets: authority support (e.g., administrative policies, additional staff), working towards common goals, cooperation, and equal group status. Pettigrew expanded the theoretical framework to include friendship building [27]. Intergenerational program participants may vary in age from preschool through university students and setting such as older adults living in long-term care facilities or independent community dwellings. Program activities also vary, potentially including art, music, and technology programs. Intergenerational technology programs existed prior to COVID-19 [28,29,30] and have been shown to produce beneficial health outcomes for both age groups, including increased physical activity, executive functioning, self-esteem, and reduced anxiety about aging [21]. Prior to COVID-19, intergenerational programs illustrated the potential to reduce social isolation and loneliness among older adults [29], but few studies specifically focused on rural communities [31].

Intergenerational programs providing technology mentoring often follow a reverse-mentoring model [20] wherein younger adults guide older adults by providing support and knowledge [32]. One example is a college student teaching an older adult how to use social media. Most intergenerational programs, including those integrating technology, are primarily conducted in person [33]. Cyber-Seniors (cyberseniors.org) is a Canadian non-profit organization founded in 2015 that partners with high schools, colleges, and universities to train students as technology mentors to older adults [34]. Cyber-Seniors aims to support older adults in remaining socially connected and self-sufficient as well as to provide meaningful opportunities for young people through a reverse-mentoring model. In a study prior to COVID-19, Breck and colleagues implemented the Cyber-Seniors program as a means of reverse mentoring young adults’ digital skills assisting older adults in increasing digital competence and social connections [29].

This study expanded on previous research of intergenerational technology programs by implementing an online version of Cyber-Seniors in a rural region of the United States during COVID-19. To our knowledge, this is the first study that utilized the Cyber-Seniors infrastructure to conduct intergenerational reverse-mentoring at a distance in a university setting. The purpose of this pilot study was to assess whether participating in an online Cyber-Seniors program with university students changed social isolation and loneliness among older adults receiving reverse-mentoring technology assistance during COVID-19.

## 2. Materials and Methods

### 2.1. Program Overview

During the fall of 2020, a university located in the southern Appalachian Mountain region of the United States partnered with the local Area Agency on Aging (AAA) to increase the social connectivity of younger and older adults as well as bridge the intergenerational divide. The AAA is a primary resource for older adults in this rural area and a central hub of resources for the seven counties making it an ideal partner to reach rural older adults. Physical distancing due to COVID-19 presented an increased need for digital literacy to provide social connectivity; however, protecting vulnerable older adults and immunocompromised adults also presented challenges in bringing generations together in person, especially in rural communities. Through the initial planning and needs assessment conversations with community partners, it became evident that there were three local community needs; (1) trained student volunteers who could serve as technology mentors to older adults, (2) older adult participants that were willing to learn and/or expand their technical skills, and (3) online programs that would serve as enrichment and recreational opportunities for homebound older adults during COVID-19. These topic areas were informed by the needs assessment conducted by the local AAA and by observations from staff in local aging organizations. Guided by the tenets of contact theory [22,27], the program gained authority support from the local AAA and the university. Then, the program focused on a common goal of improving digital literacy among older adults through a cooperative environment between university students and older adults. Program participation also created the potential for building lasting friendships.

For this study, the university partnered with the Cyber-Seniors organization to provide program support. The Cyber-Seniors organization maintains partnerships with representatives at high schools, colleges, and universities, providing them with varied service models of program delivery. Cyber-Seniors programs typically have younger, and older adults work together in person at senior centers, libraries, and community centers. However, the pandemic required physical distancing, and some programs attempted to move to an online setting. To become a technology mentor, university students completed the required mentor training and watched the Cyber-Seniors documentary. The Cyber-Seniors online self-paced training included topics such as tips for working with older adults, best practices for teaching technology to older adults, how to teach older adults to stay safe online, and how to troubleshoot technology problems. Students were required to earn a minimum of 80% proficiency on six quizzes to be considered a Cyber-Seniors mentor. In addition to the training, students watched the Cyber-Seniors documentary to learn about the history and benefits of reverse-mentoring before being matched with an older adult mentee. The study team developed support materials, communicated with students via email, and met with students outside of class to facilitate their Cyber-Seniors enrollment and training and to provide technical support. This project was designed to demonstrate its feasibility and did not have any external funding; therefore, the size and capacity were limited to what the small project team could manage, i.e., engaging a single university course as the source of mentors and recruiting as many local older adults as possible with support from community organizations.

The university Cyber-Seniors program coordinator communicated with older adults over the phone to assess the older adults’ technology needs. Then, based on university student mentors’ self-identified technology skills and availability, the university Cyber-Seniors program coordinator matched the older adult participant with a student technology mentor. The technological needs of the older adult participants ranged from learning how to find videos on YouTube, creating a spreadsheet to track personal spending, connecting a Bluetooth-enabled smart device to a car, using a new device to access Zoom meetings, and setting up an email account. Though the technology needs of the older adults ranged in levels of complexity, they were able to describe their need for mentoring in enough detail during the initial phone call assessment. This conversation enabled the university Cyber-Seniors program coordinator to summarize the individual participant’s familiarity with technology and their specific needs. Both university students and older adult participants were paired through the Cyber-Seniors system and then connected via phone calls to introduce themselves to one another and engage in the reverse-mentoring process. The student technology mentors offered solutions while on the phone or through video conferencing. Once the technology mentor identified the older adult’s technology skill level, they were often able to provide instructions and support to transition to video conferencing (i.e., Zoom). Intergenerational pairs participated in a range of 1 to 4 sessions during the 3-month pilot program, and each session had an average length of 1.5 h. This study was reviewed and classified as non-human subject research by the Institutional Review Board (IRB) at Appalachian State University (#21-0031).

### 2.2. Participants

#### 2.2.1. Older Adults

Older adult participants were recruited through the local AAA, which is connected with seven senior centers, other community organizations, and general community outreach. Using both print and digital advertisements, we distributed flyers via home-delivered meal organizations, created advertisements through the public library, and posted on social media. Senior center directors made phone calls to individual older adults to tell them about the program. Older adults registered for the program by emailing or calling the Cyber-Seniors program coordinator at the university. The recruitment process targeted individuals 50 years and older. In order to participate, older adults were required to have a telephone and a device (i.e., smartphone, laptop, printer, desktop computer). During the registration process, the university coordinator identified the needs of the older adult participant and confirmed they had an available device. Participants were invited to complete pre- and post-program surveys (described in more detail below). The survey included a statement about this study and asked for their consent to participate. Upon completion of both pre-and post-program surveys participants received one $50 gift card. Nine older adults completed the pre-survey, and eight completed the post-program survey. The average age of older adult participants was 73.5 years [range: 61–86], and they all lived in the southern Appalachian region.

#### 2.2.2. University Students

The university student participants in this study were recruited from an upper-level undergraduate gerontology course. Students could complete course requirements without participating in the Cyber-Seniors evaluation. The students were given information in class to register through the Cyber-Seniors online system. Sixty-four (64) students were enrolled in the class, and thirty-one students provided consent to participate in this study. All 31 students completed the pre-survey, and 20 completed the post-program survey. Of these, 14 students (45%) reported on the pre-survey that they worked with a local adult, and the remaining 17 (55%) reported working with an older adult they knew, most often a grandparent. In the post-survey, 11 student respondents (55%) worked with a local older adult, and 9 (45%) worked with an older adult they already knew.

### 2.3. Measures

The pre-program survey was available during the first month of the semester (August/September 2020) and the post-program survey during the last month of the semester (November/December 2020); these two time points were about 3 months apart. Participants received an email with a link to an online survey through Qualtrics. The surveys included validated measures and open-ended questions about the program and were administered through Qualtrics (see Supplementary Materials).

Both students and older adults answered questions to measure social isolation and loneliness. Respondents’ social networks—a marker for social isolation—were measured using the Lubben Social Network Scale-6, a six-item measure with two subscales that represent connections to family (3 items) and friends (3 items) [35,36,37]. Questions ask respondents to report how many people they see or hear from at least once a month, how many they feel close to and could call on for help, and how many they can talk about private matters with. Each of these questions is asked separately about relatives and about friends. There are five response options for each item: none (0), one (1), two (2), three or four (3), five to eight (4), and nine or more (5) people. Scores were summed across all six items and within each of the two subscales. Total scores can range from 0–30, and family and friend subscores can each range from 0–15, with higher scores indicating a larger network and, therefore, less isolation. We used the UCLA 3-item Loneliness Scale [38] to measure loneliness. In addition to other populations, the shortened version of the UCLA Loneliness Scale is valid and reliable (Cronbach’s Alpha = 0.89) for older adults [39] and has been used in studies with older adults [40,41]. These three items ask respondents how often they feel they lack companionship, feel left out, and feel isolated from others. Response options are hardly ever (1), some of the time (2), and often (3). The loneliness score is the sum of all three items and can range from 3–9 with higher scores representing more loneliness.

In addition to these closed-ended questions, the post-survey included 6 statements or questions developed for the evaluation that allowed participants to write in response:

1. Please tell us about your experience with this program.
2. How have your ideas about working with [young/older] adults changed or remained the same from your participation in this program? Please describe.
3. How have your interactions with others changed during the current COVID situation?
4. Please describe if your participation in this program provided any additional opportunities for social interaction and how that made you feel.
5. Please describe any specific interactions through this program that influenced your ideas of [young/older] adults.
6. Why would or wouldn’t you recommend the Cyber-Seniors program?

### 2.4. Analysis

This study utilized surveys with both quantitative (defined response options) and qualitative (open-ended responses) data to address the study purpose. We used a triangulation of methods by simultaneously gathering qualitative and quantitative data to be more comprehensive and gain a richer understanding of the participants’ perspectives [42]. For defined response questions, we calculated and compared mean scores and standard deviations on the isolation and loneliness scales during the pre- and post-program periods for older and younger adult participants. Although we did not anticipate adequate statistical power to make comparisons *a priori* given the inclusion of a single course in the pilot, we conducted *t*-tests of the differences between pre- and post-program surveys. We used a conservative alpha value of 0.20 to indicate statistical differences given the planned small sample size. Our primary analyses included all respondents at each point in time (pre and post). As a sensitivity analysis, we included only respondents who answered both the pre- and post-surveys (linked by participant ID).

Analysis of the open-ended questions was guided by directed content analysis [43]. The structured approach of directed content analysis was more suitable for this study than other approaches because of prior research on our *a priori* domains of focus (i.e., loneliness and social isolation). Aligned with the definitions of the study domains, three authors (J.J.J., E.D.B., K.U.) coded responses with isolation and loneliness as predetermined codes. To study trustworthiness, we had three authors independently read and code the participants’ open-ended responses. Then, the three authors met to reach a consensus on which responses represented the domains of interest. Three additional codes (i.e., learned something new, general program feedback, digital divide) were also identified that were not applicable to this study. Exemplar open-ended responses to the domains of interest are provided as descriptive evidence to support the quantitative findings [43].

## 3. Results

### 3.1. Older Adult Experiences

At baseline, older adults’ mean score on the Lubben Social Network Scale-6 was 19.6 (SD = 7.3; Table 1). After participating in the program, it increased to 21.4 (SD = 7.1), on average, indicating lower social isolation at the later time point, though this difference was not statistically significant (*p* = 0.61). Subscale scores also did not change significantly, but mean scores increased, on average, at follow-up in both the family (9.6 [SD = 4.1] to 10.9 [SD = 3.4], *p* = 0.48) and friends (10.0 [SD = 4.0] to 10.5 [SD = 4.6], *p* = 0.81) domains. Comments from older adults in the open-ended questions supported the notion that isolation decreased because of skills developed in the program. In response to the question about COVID-19, many reported that they had separated themselves from family and friends because of concerns about illness, often because of their underlying health conditions. One participant noted that COVID-19 had created both isolation and feelings of loneliness: “COVID has changed everything, nothing is the same. I am isolated, mask[s] are uncomfortable so I just stay home. Unless I go to the doctor. Not seeing my family is devastating. (#108)” The Cyber-Seniors program provided older adults with an opportunity to expand their network or reconnect with family and friends using technology. As one participant said, “I was able to interact with my Rotary Club on a weekly basis through Zoom, which Miss [student name] taught me how to do. I am very grateful to her for this, and grateful to you too for this program. (#102)”.

Loneliness was lower among older adult participants at the post-program time point than at baseline, and this difference was statistically significant (*p* = 0.17). The mean score on the UCLA three-item Loneliness Scale was 5.6 (SD = 2.2) at baseline and 4.1 (SD = 1.3) in the follow-up survey. Several participants indicated in their comments that the Cyber-Seniors program alleviated some of their feelings of loneliness by offering something to look forward to in their days while staying at home. For example, one said, “I found myself looking forward to [student name]’s call on Tuesday at 11:30 am. As I use the technology I learned from these sessions I catch myself saying ’thank you, [student name].’ Guess I have made a lasting friend. (#101)” Others indicated that program participation helped them stay connected virtually during the pandemic using social media and web-based communication tools.

### 3.2. University Student Experiences

Unlike older adults, students reported more social isolation at follow-up than at baseline: mean scores on the Lubben Social Network Scale-6 went from 19.6 (SD = 4.3) to 18.0 (SD = 4.1); this difference was not statistically significant (*p* = 0.20). Students did report significantly fewer connections in the family domain (9.4 [SD = 3.0] to 8.1 [SD = 2.8], *p* = 0.14) over the course of the program. Scores also declined, on average, in the friends domain (10.3 [SD = 2.7] to 9.9 [SD = 2.7]), but not significantly (*p* = 0.69). Mean loneliness scores increased slightly, but not significantly (*p* = 0.73), among students, from 5.0 (SD = 1.5) on the baseline survey to 5.2 (SD = 1.7) on the post-program survey.

### 3.3. Sensitivity Analyses

When we included only respondents who answered both pre- and post-surveys, the results were similar to those reported above. Specifically, among the five older adults who completed the Lubben Social Network Scale-6 at both time points, the mean overall social network score increased from 20.4 (SD = 8.4) [range: 6–27] to 22.8 (SD = 7.5) [range: 10–28], but the difference was not statistically significant (*p* = 0.65). Mean scores also increased in both the family and friends domains: 10.0 (SD = 3.3) [range: 5–13] to 11.8 (SD = 3.0) [range: 7–15] in the family domain and 10.4 (SD = 5.4) [range: 1–14] to 11.0 (SD = 4.6) [range: 3–14] in the friends domain. Neither of these was statistically significant (*p* = 0.40 for family and *p* = 0.85 for friends). Two older adults did not answer the loneliness items at baseline. Among the remaining three, the mean loneliness score declined from 6.0 (SD = 2.0) [range: 3–6] at baseline to 4.0 (SD = 1.2) [range: 4–8] at follow-up, which was statistically significant by our criteria (*p* = 0.12) and consistent with the full sample result.

Ten students could be linked in the pre- and post-survey. Similar to the full group of student participants, isolation among these 10 students increased between the two time points with scores declining from 20.0 (SD = 4.5) [range:10–26] to 19.0 (SD = 4.8) [range: 12–29], on average. These declines occurred in both family (9.5 (SD = 4.8) [range: 3–13] to 9.2 (SD = 2.9) [range: 4–14]) and friends (10.5 (SD = 2.9) [range: 6–14] to 9.8 (SD = 8.1) [range: 6–15]) domains. None of these differences were statistically significant (*p* = 0.63 overall, *p* = 0.83 for family, and *p* = 0.56 for friends). Loneliness also increased slightly among these 10 students, from 4.8 (SD = 1.8) [range: 3–8] at baseline to 5.0 (SD = 1.3) [range: 3–7] at follow-up, though the difference was not statistically significant (*p* = 0.78).

## 4. Discussion

This fully online adaptation of the Cyber-Seniors mentoring program suggests that it is effective for older adults in Appalachia in reducing loneliness. While we did not see significant differences in social isolation, the results indicate a possible benefit in that domain as well, as students experienced a reduction in family connections over the semester while older adults did not. Findings from this study suggest existing intergenerational technology mentoring programs can successfully transition to an online delivery format and that they may serve as a mechanism for social change, decreasing loneliness among participating older adults [2]. These changes are notable given that they occurred during the first year of COVID-19 (August–December 2020), when people involved in the study were experiencing many changes in their lives and routines.

Previous Cyber-Seniors research focused on outcomes resulting from in-person program implementation [20,29,38,44]. Findings from this adapted fully online model were consistent with Breck and colleagues’ qualitative findings that program participation increased connection [29]. In addition to the quantitative findings of this study, older adults’ qualitative comments also illustrated that they acquired new skills and confidence to use digital tools, thereby enabling them to increase connectivity with others and preventing loneliness. COVID-19 accelerated online program implementation that may address social isolation and loneliness [45]. While these pilot results are encouraging, the challenges of implementing an online technology program were substantial. The program required personnel who had the skills to assist student technology mentors from a distance and who could devote time to addressing technical challenges encountered by students and older adults alike. Future studies should examine the implementation process in diverse contexts, including program components, delivery platforms, classroom context, and university characteristics.

This study had limitations, including small sample size and the lack of in-depth qualitative data. Recruitment of local older adults was a challenge, potentially because of a lack of resources for outreach but also possibly because of skepticism or uncertainty of online interaction on the part of many older adults in the community. Finally, the results from our region of Appalachia may not generalize to older adults in all rural areas. There is also the potential that observed differences could result from different individuals responding to the pre- and post-surveys rather than changes during the program period. Our sensitivity analyses suggest that this did not occur in the pilot and that respondents at both time points were relatively similar. Older adults and students who completed surveys at both time points reported less isolation than their peers who completed only a single time point, on average. This does suggest that non-respondents may have been more isolated and lonelier than respondents, but we do not have data to evaluate whether this impacted the program’s effect. Future studies that are larger will be needed to test whether this fully online adaptation of Cyber-Seniors is effective in reducing isolation and loneliness among older adults in rural areas. Additional work in both rural and urban settings and studies that include both in-person and fully online options will be helpful in understanding whether online intergenerational programs can reduce disparities in rural and other areas with limited resources. Future studies could benefit from enrolling older adults in person and then shifting to an online or hybrid option.

As rural Appalachian areas increase access to broadband [2,19], there will be an increased demand for partnerships to build community capacity for addressing the digital needs of older adults. In rural Appalachian communities, older adults face economic disadvantages, a greater risk of chronic disease, and limited access to the internet and devices [46,47]. Although this study was conducted during COVID-19 physical distancing restrictions, rural communities historically have struggled to provide services to residents due to physical distance from large healthcare institutions and service providers and limited staff [48]. Intergenerational technology programs (e.g., Cyber-Seniors) provide organizations with the infrastructure to recruit, train, schedule, and evaluate programs. By increasing digital access and technology skills, rural older adults would have more support services available. Adding an online option along with in-person services can expand access for older adults who are homebound or have health conditions or impairments that make it difficult for them to attend in-person programming. Aligned with contact theory [22,27], authority support at the local level is essential and requires strong partnerships between organizations such as Area Agencies on Aging and high schools/colleges/universities. It would behoove future studies to collaborate with local champions to increase buy-in and ease the concerns of older adults who are hesitant to ask for technology assistance in a fully online program.

## 5. Conclusions

This pilot study suggests that an online reverse mentoring program improves social outcomes for older adults in rural areas or when in-person contact is not possible. Further studies are needed to validate these findings. Intergenerational technology mentoring programs have the potential to decrease loneliness and social isolation because they support positive social connections between older adults and university students while enhancing skills. These social connections, in turn, may improve health among older adults.

## Figures and Tables

**Table 1:** Mean social network and loneliness scores among older adults and students participating in the Cyber-Seniors program, August–December 2020.

Measure	Older Adults			Students		
	Baseline Mean (SD) (Range) *n*	Follow-Up Mean (SD) (Range) *n*	*p*-Value ^1^	Baseline Mean (SD) (Range) *n*	Follow-Up Mean (SD) (Range) *n*	*p*-Value ^1^
Social isolation (Lubben Social Network Scale-6; Total)	19.6 (7.3)	21.4 (7.1)	0.61	19.6 (4.3)	18.0 (4.1)	0.20
	(6–27)	(10–28)		(10–30)	(12–29)	
	*n* = 9	*n* = 8		*n* = 30	*n* = 18	
Family domain	9.6 (4.1)	10.9 (3.4)	0.48	9.4 (3.0)	8.1 (2.8)	0.14
	(3–14)	(7–15)		(3–15)	(3–14)	
	*n* = 9	*n* = 8		*n* = 30	*n* = 18	
Friends domain	10.0 (4.0)	10.5 (4.6)	0.81	10.3 (2.7)	9.9 (2.7)	0.69
	(1–14)	(3–15)		(5–15)	(5–15)	
	*n* = 9	*n* = 8		*n* = 30	*n* = 18	
UCLA 3-item Loneliness Scale	5.6 (2.2)	4.1 (1.3)	0.17	5.0 (1.5)	5.2 (1.7)	0.73
	(3–9)	(3–6)		(3–8)	(3–9)	
	*n* = 7	*n* = 7		*n* = 30	*n* = 18	

Note: Higher scores on the social isolation measure indicate a larger network and, therefore, less isolation. Conversely, higher scores on the loneliness measures indicate more loneliness. ^1^ *p*-value based on a *t*-test comparing baseline and follow-up within the group (e.g., baseline and follow-up for older adults).

## Data Availability

The data presented in this study are available on request from the corresponding author. The data are not publicly available due to the pilot nature of the data and the small sample, which potentially could compromise confidentiality.

## Supplementary Materials

The following supporting information can be downloaded at: https://www.mdpi.com/article/10.3390/ijerph19127121/s1. References [20,37,49,50,51,52,53] are cited in the Supplementary Materials.

## References

[1] Holt-Lunstad J., Smith T.B., Layton J.B. (2010). Social Relationships and Mortality Risk: A Meta-analytic Review. PLoS Med..

[2] Rachfal C.L. (2021). The Digital Divide: What Is It, Where Is It, and Federal Assistance Programs.

[3] Perissinotto C.M., Stijacic Cenzer I., Covinsky K.E. (2012). Loneliness in Older Persons. Arch. Intern. Med..

[4] Lenz Lock S., Chura L.R. (2017). Global Council on Brain Health: Survey Data and Recommendations Highlight Opportunities to Promote Physical Activity, Sleep, and Social Engagement in Adults. Alzheimer’s Dement..

[5] DiNapoli E.A., Wu B., Scogin F. (2013). Social Isolation and Cognitive Function in Appalachian Older Adults. Res. Aging.

[6] Lubben J., Gironda M., Berkman B., Harootyan L. (2003). Centrality of Social Ties to the Health and Well-Being of Older Adults. Social Work and Health Care in an Aging Society: Education, Policy, Practice, and Research.

[7] Perlman D., Peplau L.A. (1981). Toward a Social Psychology of Loneliness. Personal Relationships.

[8] Coleman J.S. (1988). Social Capital in the Creation of Human Capital. Am. J. Sociol..

[9] Wong J.S., Waite L.J., Bengston V.L., Gans D., Putney N.M., Silverstein M. (2016). Theories of Social Connectedness and Aging. Handbook of Theories of Aging.

[10] Holt-Lunstad J. (2017). The Potential Public Health Relevance of Social Isolation and Loneliness: Prevalence, Epidemiology, and Risk Factors. Public Policy Aging Rep..

[11] Killgore W.D.S., Cloonan S.A., Taylor E.C., Dailey N.S. (2020). Loneliness: A Signature Mental Health Concern in the Era of COVID-19. Psychiatry Res..

[12] Kotwal A.A., Holt-Lunstad J., Newmark R.L., Cenzer I., Smith A.K., Covinsky K.E., Escueta D.P., Lee J.M., Perissinotto C.M. (2020). Social Isolation and Loneliness among San Francisco Bay Area Older Adults during the COVID-19 Shelter-in-Place Orders. J. Am. Geriatr. Soc..

[13] O’Sullivan R., Burns A., Leavey G., Leroi I., Burholt V., Lubben J., Holt-Lunstad J., Victor C., Lawlor B., Vilar-Compte M. (2021). Impact of the COVID-19 Pandemic on Loneliness and Social Isolation: A Multi-Country Study. Int. J. Environ. Res. Public Health.

[14] Teater B., Chonody J.M., Hannan K. (2021). Meeting social needs and loneliness in a time of social distancing under COVID-19: A comparison among young, middle, and older adults. J. Hum. Behav. Soc. Environ..

[15] Clare C.A. (2021). Telehealth and the Digital Divide as a Social Determinant of Health during the COVID-19 Pandemic. Netw. Model. Anal. Health Inform. Bioinform..

[16] Hunsaker A., Hargittai E. (2018). A Review of Internet Use among Older Adults. New Media Soc..

[17] Van Jaarsveld G.M. (2020). The Effects of COVID-19 Among the Elderly Population: A Case for Closing the Digital Divide. Front. Psychiatry.

[18] Anderson M., Vogels E.A. (2020). Americans Turn to Technology During COVID-19 Outbreak, Say An Outage Would Be a Problem.

[19] Pollard K., Jacobsen L.A. (2021). The Appalachian Region: A Data Overview from the 2015–2019 American Community Survey Chartbook.

[20] Leedahl S.N., Brasher M.S., Estus E., Breck B.M., Dennis C.B., Clark S.C. (2018). Implementing an interdisciplinary intergenerational program using the Cyber Seniors^®^ reverse mentoring model within higher education. Gerontol. Geriatr. Educ..

[21] Generations United (2018). All in Together: Creating Places Where Young and Old Thrive.

[22] Allport G. (1954). The Nature of Prejudice.

[23] Imperato C., Schneider B.H., Caricati L., Amichai-Hamburger Y., Mancini T. (2021). Allport meets internet: A meta-analytical investigation of online intergroup contact and prejudice reduction. Int. J. Intercult. Relat..

[24] Jarrott S.E., Smith C.L. (2011). The Complement of Research and Theory in Practice: Contact Theory at Work in Nonfamilial Intergenerational Programs. Gerontologist.

[25] Kuehne V.S., Melville J. (2014). The State of Our Art: A Review of Theories Used in Intergenerational Program Research (2003–2014) and Ways Forward. J. Intergenerational Relatsh..

[26] Weaver R.H., Naar J.J., Jarrott S.E. (2019). Using Contact Theory to Assess Staff Perspectives on Training Initiatives of an Intergenerational Programming Intervention. Gerontologist.

[27] Pettigrew T.F. (1998). Intergroup Contact Theory. Annu. Rev. Psychol..

[28] Bernard M.M., Fruhwirth M., Brooks M., Oakley K., Wang X., Ouechni K.G., Janson F. (2011). Intergenerational Telementoring for the Promotion of Social Relationships. Gerontechnology.

[29] Breck B.M., Dennis C.B., Leedahl S.N. (2018). Implementing Reverse Mentoring to Address Social Isolation among Older Adults. J. Gerontol. Soc. Work.

[30] Kolodinsky J., Cranwell M., Rowe E. (2002). Bridging the Generation Gap Across the Digital Divide: Teens Teaching Internet Skills to Senior Citizens. J. Ext..

[31] Bullock J.R., Osborne S.S. (1999). Seniors’, Volunteers’, and Families’ Perspectives of an Intergenerational Program in a Rural Community. Educ. Gerontol..

[32] Murphy W.M. (2012). Reverse mentoring at work: Fostering cross-generational learning and developing millennial leaders. Hum. Resour. Manag..

[33] Canedo-García A., García-Sánchez J.-N., Pacheco-Sanz D.-I. (2017). A Systematic Review of the Effectiveness of Intergenerational Programs. Front. Psychol..

[34] Cyber-Seniors-Cyber-Seniors Inc. https://cyberseniors.org/.

[35] Gray J., Kim J., Ciesla J.R., Yao P. (2014). Rasch Analysis of the Lubben Social Network Scale–6 (LSNS-6). J. Appl. Gerontol..

[36] Jang Y., Powers D.A., Park N.S., Chiriboga D.A., Chi I., Lubben J. (2022). Performance of an Abbreviated Lubben Social Network Scale (LSNS-6) in Three Ethnic Groups of Older Asian Americans. Gerontologist.

[37] Lubben J., Blozik E., Gillmann G., Iliffe S., von Renteln Kruse W., Beck J.C., Stuck A.E. (2006). Performance of an Abbreviated Version of the Lubben Social Network Scale Among Three European Community-Dwelling Older Adult Populations. Gerontologist.

[38] LoBuono D., Leedahl S., Maiocco E. (2019). Older adults learning technology in an intergenerational program: Qualitative analysis of areas of technology requested for assistance. Gerontechnology.

[39] Russell D.W. (1996). UCLA Loneliness Scale (Version 3): Reliability, Validity, and Factor Structure. J. Pers. Assess..

[40] Ong A.D., Uchino B.N., Wethington E. (2015). Loneliness and Health in Older Adults: A Mini-Review and Synthesis. Gerontology.

[41] Penning M.J., Liu G., Chou P.H.B. (2013). Measuring Loneliness Among Middle-Aged and Older Adults: The UCLA and de Jong Gierveld Loneliness Scales. Soc. Indic. Res..

[42] Neuman W.L., William L. (2014). Social Research Methods: Qualitative and Quantitative Approaches.

[43] Hsieh H.-F., Shannon S.E. (2005). Three Approaches to Qualitative Content Analysis. Qual. Health Res..

[44] Leedahl S.N., Brasher M.S., LoBuono D.L., Wood B.M., Estus E.L. (2020). Reducing Ageism: Changes in Students’ Attitudes after Participation in an Intergenerational Reverse Mentoring Program. Sustainability.

[45] Gorenko J.A., Moran C., Flynn M., Dobson K., Konnert C. (2020). Social Isolation and Psychological Distress Among Older Adults Related to COVID-19: A Narrative Review of Remotely-Delivered Interventions and Recommendations. J. Appl. Gerontol..

[46] Coburn A.F., Ziller E., Paluso N., Thayer D., Talbot J.A. (2019). Long-Term Services and Supports Use Among Older Medicare Beneficiaries in Rural and Urban Areas. Res. Aging.

[47] Hash K.M., Jurkowski E.T., Krout J.A., Hash K.M., Jurkowski E.T., Krout J.A. (2015). Conclusions and Future Directions. Aging in Rural Places: Policies, Programs, and Professional Practice.

[48] Weaver R.H., Roberto K.A. (2021). Location Matters: Disparities in the Likelihood of Receiving Services in Late Life. Int. J. Aging Hum. Dev..

[49] Laidlaw K., Power M.J., Schmidt S., WHOQOL-OLD Group (2007). The Attitudes to Ageing Questionnaire (AAQ): Development and psychometric properties. Int. J. Geriatr. Psychiatry..

[50] Lasher K.P., Faulkender P.J. (1993). Measurement of aging anxiety: Development of the Anxiety about Aging Scale. Int. J. Aging Hum. Dev..

[51] Chen G., Gully S.M., Eden D. (2016). Validation of a New General Self-Efficacy Scale. Organ. Res. Methods.

[52] Glass T.A., De Leon C.F.M., Bassuk S.S., Berkman L.F. (2006). Social engagement and depressive symptoms in late life: Longitudinal findings. J. Aging Health.

[53] Hughes M.E., Waite L.J., Hawkley L.C., Cacioppo J.T. (2004). A Short Scale for Measuring Loneliness in Large Surveys. Res. Aging.

