# Supplementary material for: Virtual Intergenerational Reverse-Mentoring Program Reduces Loneliness among Older Adults: Results from a Pilot Evaluation
Source: Int J Environ Res Public Health. 2022 Jun 10;19(12):7121. doi: 10.3390/ijerph19127121 (PMC9222564; doi:10.3390/ijerph19127121)
Supplement: Supplementary file 1 [file ijerph-19-07121-s001.zip › ijerph-1637074-supplementary.pdf]

Older Adult Survey
#1-8. Psychological Growth sub-scale from the **Attitudes Toward Aging Scale**[49] ; sum to create score ranging from 8-40 with higher scores indicating greater embrace of growing older.

#9-13. Psychological Concerns sub-scale (5 items) from the **Anxiety about Aging scale**[50]; sum to create score ranging from 5-25 with higher scores indicating less anxiety.

#14-21. **New General Self-Efficacy Scale** (8 items)[51]; sum to create score ranging from 8-40 with higher scores indicating greater self-efficacy.

#22-27. **Lubben Social Network Scale** (LSNS-6)[37]; sum to create score ranging from 0-30 with higher scores indicating less isolation. Family subscale is constructed from the three LSNS-6 items that ask about relatives. Similarly, Friends subscale is constructed from the three items that ask about friends.

#28-41. Social engagement measure derived from **Glass, Mendes de Leon, Bassuk, & Berkman**[52]; sum scores (see points below for yes/no questions) ranging from 0-27 with higher scores indicating more social engagement. Set score to missing if three or more of the component questions were missing. Sub-scales for productive activities (6 items), social/leisure activities (5 items), and physical activity (3 items).

#42-44. **UCLA three-Item Loneliness Scale**[53], scored as simple sum of items ranging from 3-9 with higher scores reflecting more loneliness.

#45-47. Questions about attitudes about technology from the **original CyberSeniors article**[20]. Scores are sum of items ranging from 3-15 with higher scores representing more comfort with technology.

#48-50. Questions about working with younger adults from the **original CyberSeniors article**[20]. Scores are sum of items ranging from 0-12 with higher scores representing more likelihood of or confidence working with older adults in the future.

The following items were only asked on the post-survey. Items 1-50 were asked on both pre- and post-surveys.

#51. How likely is it that you would recommend this program to a friend or colleague?

[0-10 slider scale with labels at 1 “not at all likely,” 5 “neutral,” and 10 “extremely likely] This is the **Net Promoter Score**.

52. Please tell us about your experience with this program.

53. How have your ideas about working with [young/older] adults changed or remained the same from your participation in this program? Please describe.

54. How have your interactions with others changed during the current COVID situation?

55. Please describe if your participation in this program provided any additional opportunities for social interaction and how that made you feel.

56. Please describe any specific interactions through this program that influenced your ideas of young adults.

57. Why would or wouldn't you recommend the Cyber-Seniors program?

Student Survey
#1-6. Psychological Growth sub-scale from the **Attitudes Toward Aging Scale**[49]; modified to exclude items aimed at people who are already older. Sum to create score ranging from 6-30 with higher scores indicating greater embrace of growing older.

#7-16. Fear of Older People sub-scale (5 items) from the **Anxiety about Aging scale**[50]; sum to create score ranging from 5-25 with higher scores indicating less anxiety toward older people. Psychological Concerns sub-scale (5 items) from the Anxiety about Aging scale[50]; sum to create score ranging from 5-25 with higher scores indicating less anxiety.

#17-24. **New General Self-Efficacy Scale** (8 items)[51]; sum to create score ranging from 8-40 with higher scores indicating greater self-efficacy.

#25-30. **Lubben Social Network Scale** (LSNS-6)[37]; sum to create score ranging from 0-30 with higher scores indicating less isolation. Family subscale is constructed from the three LSNS-6 items that ask about relatives. Similarly, Friends subscale is constructed from the three items that ask about friends.

#31-44. Social engagement measure derived from **Glass, Mendes de Leon, Bassuk, & Berkman**[52]; sum scores (see points below for yes/no questions) ranging from 0-27 with higher scores indicating more social engagement. Set score to missing if three or more of the component questions were missing. Sub-scales for productive activities (6 items), social/leisure activities (5 items), and physical activity (3 items).

#45-47. **UCLA three-Item Loneliness Scale**[53], scored as simple sum of items ranging from 3-9 with higher scores reflecting more loneliness.

#48-50. Questions about working with older adults from the **original CyberSeniors article**[20]. Scores are sum of items ranging from 0-12 with higher scores representing more likelihood of or confidence working with older adults in the future.

The following items were only asked on the post-survey. Items 1-50 were asked on both pre- and post-surveys.

51. Please tell us about your experience with this program.

52. How have your ideas about working with [young/older] adults changed or remained the same from your participation in this program? Please describe.

53. How have your interactions with others changed during the current COVID situation?

54. Please describe if your participation in this program provided any additional opportunities for social interaction and how that made you feel.

55. Please describe any specific interactions through this program that influenced your ideas of older adults.

56. Why would or wouldn't you recommend the Cyber-Seniors program?
